# Research on the Multiple Small Target Detection Methodology in Remote Sensing

**DOI:** 10.3390/s24103211

**Published:** 2024-05-18

**Authors:** Changman Zou, Wang-Su Jeon, Sang-Yong Rhee

**Affiliations:** 1 Department of IT Convergence Engineering, University of Kyungnam, Changwon 51767, Republic of Korea; zouchangman@beihua.edu.cn; 2 College of Computer Science and Technology, Beihua University, Jilin 132013, China; 3 Department of Computer Engineering, University of Kyungnam, Changwon 51767, Republic of Korea; jws2218@naver.com

**Keywords:** YOLOv7, remote sensing image, target detection, MFE, DP-MLP, SSLM

## Abstract

This study focuses on advancing the field of remote sensing image target detection, addressing challenges such as small target detection, complex background handling, and dense target distribution. We propose solutions based on enhancing the YOLOv7 algorithm. Firstly, we improve the multi-scale feature enhancement (MFE) method of YOLOv7, enhancing its adaptability and precision in detecting small targets and complex backgrounds. Secondly, we design a modified YOLOv7 global information DP-MLP module to effectively capture and integrate global information, thereby improving target detection accuracy and robustness, especially in handling large-scale variations and complex scenes. Lastly, we explore a semi-supervised learning model (SSLM) target detection algorithm incorporating unlabeled data, leveraging information from unlabeled data to enhance the model’s generalization ability and performance. Experimental results demonstrate that despite the outstanding performance of YOLOv7, the mean average precision (MAP) can still be improved by 1.9%. Specifically, under testing on the TGRS-HRRSD-Dataset, the MFE and DP-MLP models achieve MAP values of 93.4% and 93.1%, respectively. Across the NWPU VHR-10 dataset, the three models achieve MAP values of 93.1%, 92.1%, and 92.2%, respectively. Significant improvements are observed across various metrics compared to the original model. This study enhances the adaptability, accuracy, and generalization of remote sensing image object detection.

## 1. Introduction

The field of remote sensing satellite imagery is distinguished by its remarkable high resolution and rapid data acquisition capabilities, which impose elevated demands on image processing techniques. Consequently, the swift and accurate extraction of features from remote-sensing images has evolved into a pivotal technology within this domain. The advent of target detection in remote sensing imagery, bolstered by advancements in remote sensing technology, offers expansive coverage, long-range observation, and high operational efficiency. These advancements have profound implications for both military and civilian applications. In military contexts, real-time monitoring applications of remote sensing images enable the tracking and targeting of small moving objects on the ground. Conversely, in the civilian sector, remote sensing is utilized in disaster assessment [1,2], environmental monitoring [3,4], urban planning [5,6], surveying and mapping [7,8], and resource exploration [9,10], among other areas critical to public welfare.

Remote sensing image target detection and recognition constitute fundamental tasks in optical remote sensing image processing. Remote sensing images present challenges such as wide field of view, high background complexity, unique perspectives, target rotation, and small target sizes, posing significant challenges for target detection tasks [11,12,13,14,15]. The objective of remote sensing target detection is to determine the presence of objects in an image, locate them accurately, and then classify them.

With the rapid development of remote sensing technology, there is an increasing number of remote sensing detectors available. Consequently, the volume of remote sensing image data is also increasing, with high-resolution multispectral remote sensing images becoming more prevalent [16]. This influx of remote sensing image data provides abundant resources but also presents new challenges, particularly in efficiently and accurately detecting and recognizing objects within the data, which is currently a hot topic in remote sensing image processing [17,18,19]. However, inherent sensor characteristics such as physical technology properties, observation angles, and imaging mechanisms introduce unavoidable noise into collected remote sensing images. Additionally, external interferences such as weather conditions, cloud cover, lighting variations, and object colors further affect target detection tasks in various environments [20].

In summary, remote sensing images pose several challenges for target detection tasks compared to conventional images due to their wide coverage, high resolution, and complex backgrounds. These challenges include the following:

Diverse target scales: Remote sensing images span a wide range of scales, leading to variations in the size of the same target across different images.

Small target detection: Small objects occupy a small proportion of the image and after feature extraction and they may be further reduced, leading to instances of missed detections.

Dense scene interference: Overlapping objects in dense scenes can cause repetitive target selection errors due to the reduced detection performance of current remote sensing image detection networks.

Complex target arrangements: The complex arrangement of objects in images also poses challenges for detection, hindering the accurate determination of target orientations.

The remarkable performance of deep convolutional neural networks in image processing has garnered increasing attention. As deep learning techniques advance, their application in target detection and recognition tasks within remote sensing images becomes a popular trend. Compared to traditional detection methods, deep learning algorithms automatically extract target features, saving a significant amount of human effort in feature design. Moreover, deep learning algorithms often achieve better and more accurate feature extraction compared to manually designed features, making them more representative in certain scenarios [21,22,23].

In the field of remote sensing images, traditional target detection algorithms based on manually designed features are similar to those used in conventional images. The traditional target detection process involves candidate region extraction, feature extraction, classifier design, and post-processing. Initially, potential target regions are extracted from the input image using candidate region extraction methods. Subsequently, features are extracted for each region, followed by classification based on the extracted features. Finally, post-processing filters and merges the obtained candidate boxes to produce the final results. Commonly used features in remote sensing target detection include color features [24,25], texture features [26], edge shape features [27], and contextual information. Since a single feature may not adequately represent the characteristics of the target, multiple-feature fusion is often employed. Remote sensing image processing based on neural networks is mostly transplanted from algorithms in the natural image processing domain and then optimized for the characteristics of remote sensing images. The target detection task based on deep learning directly employs convolutional neural networks to complete the process, including feature extraction, multi-category classification of targets, and target localization. It can handle and obtain primary texture and high-level semantic features of images. Through training and learning with large amounts of data, it automatically completes the target detection task, without the need for manual processing of massive amounts of data. With the continuous development of remote sensing technology and the popularization of low-altitude unmanned aerial vehicles, remote sensing image acquisition has become more convenient and efficient.

In the field of deep learning-based remote sensing image target detection algorithms, algorithm improvement is a critical research direction aimed at enhancing detection accuracy, speed, and the ability to detect small or sparse targets in complex backgrounds. The following are some detailed aspects of algorithm improvement.

Optimization of Convolutional Neural Networks (CNNs) [28]: Enhancing feature extraction capabilities by designing deeper or more complex network structures. For instance, introducing residual connections or dense connections can reduce information loss during training, thereby improving the network’s learning ability [29,30].

Improvements in the Region-Based Convolutional Neural Network (R-CNN) [31] Series: This includes Fast R-CNN [32], Faster R-CNN [33], etc., which aim to improve the speed and accuracy of target detection by enhancing the region proposal mechanism. For example, Faster R-CNN introduces a Region Proposal Network (RPN) to achieve fast and efficient extraction of target candidate regions.

Optimization of Single-Shot Detectors (e.g., YOLO [34], SSD [35]): These algorithms detect targets by scanning the image once, focusing on improving processing speed and detection accuracy. Further performance enhancements can be achieved by improving loss functions, network architectures, or introducing new regularization techniques.

Addressing the Special Challenges of Remote Sensing Images: Target detection in remote sensing images faces challenges such as different scales, rotations, and occlusions. Researchers improve model robustness and accuracy by introducing scale-invariant and rotation-invariant network architectures or utilizing multi-scale feature fusion techniques.

Application of Attention Mechanisms: Attention mechanisms help models focus on key information in the image, thereby improving detection accuracy [36,37,38].

Among various deep learning techniques, YOLOv7 [39] has emerged as a popular choice due to its real-time processing capabilities and high detection accuracy. However, challenges such as detecting small targets, handling complex backgrounds, and dealing with densely distributed objects remain significant hurdles in remote sensing image target detection.

Several studies have attempted to address these challenges but there is still room for improvement. The current state-of-the-art methods often focus on specific aspects of target detection, leading to suboptimal performance in real-world scenarios. Moreover, the effectiveness of existing approaches may vary depending on the characteristics of the remote-sensing images and the nature of the targets.

To overcome these challenges and further improve the performance of YOLOv7 in remote sensing image target detection, novel approaches need to be explored. These approaches could include the following:

Enhanced Feature Fusion: Developing advanced feature fusion techniques to better integrate features from different network layers, thereby improving the model’s ability to detect small targets and handle complex backgrounds [40,41].

Global Information Integration: Designing innovative modules to effectively capture and integrate global information in YOLOv7, enhancing detection accuracy and robustness, particularly in scenarios with large-scale variations and complex scenes [42,43].

Semi-Supervised Learning: Exploring semi-supervised learning algorithms that leverage large amounts of unlabeled remote sensing image data to enhance the model’s generalization ability and performance, offering an effective solution for target detection under resource-constrained situations [44,45].

By incorporating these advancements into YOLOv7-based algorithms, researchers can contribute to the ongoing progress in remote sensing image target detection, addressing the existing challenges and advancing the capabilities of deep learning techniques in this field.

In this context, this paper proposes an improved YOLOv7-based method for remote sensing image target detection. By enhancing the multi-scale feature extraction capabilities of YOLOv7 and incorporating advanced techniques for handling complex backgrounds, we aim to achieve superior performance in detecting small targets and accurately localizing objects in densely populated scenes. Additionally, we explore the integration of semi-supervised learning techniques to leverage unlabeled data and improve the model’s generalization ability.

Through comprehensive experimentation and comparative analysis, we demonstrate the effectiveness of our proposed method in addressing the aforementioned challenges. Our results highlight the importance of adapting deep learning techniques to the specific requirements of remote sensing applications. By advancing the state-of-the-art techniques in remote sensing image target detection, our work contributes to the broader goal of harnessing the potential of deep learning for real-world applications in geospatial analysis and environmental monitoring.

## 2. Related Works

### 2.1. Network Structure Optimization

In the domain of remote sensing image target detection, the optimization of network architecture is crucial for enhancing the accuracy, efficiency, and robustness of algorithms. Innovations in this field have been focused on several key strategies, including the introduction of efficient network architectures, deep feature fusion, the application of attention mechanisms, the incorporation of Transformer structures, and adaptive and dynamic structure adjustments. These strategies aim to address the diverse challenges posed by remote sensing imagery, such as the need for real-time processing, the detection of targets across varying dimensions, and the accurate classification and localization of objects within complex scenes.

Efficient Network Architectures: The deployment of lightweight network architectures like MobileNet [46] and ShuffleNet [47] has significantly reduced parameter count and computational complexity, thus accelerating target detection processes while maintaining high performance. Additionally, modifications to deep network structures such as ResNet [48] and DenseNet [49] have improved feature extraction efficiency and effectiveness for remote sensing characteristics.

Deep Feature Fusion: Techniques like Feature Pyramid Networks (FPN) enhance detection capabilities for targets of different sizes by combining high-level semantic features with low-level detail features [50]. This approach is beneficial for capturing characteristics of both large and small-scale targets, improving detection accuracy and recall rates.

Attention Mechanisms: Spatial and channel attention mechanisms, exemplified by Squeeze-and-Excitation (SE) [51] modules and Convolutional Block Attention Module (CBAM) [52], focus the network’s attention on crucial parts of the image, thereby boosting the precision and robustness of remote sensing image target detection.

Transformer Structures: The adoption of Vision Transformer architectures aids in capturing global dependencies, enhancing detection accuracy and generalization capability through its adeptness in processing sequential data [53].

Adaptive and Dynamic Adjustments: Dynamic convolution adjusts convolution kernel parameters, enabling the network to adapt its feature extraction methods to the input image’s characteristics. This adaptability is essential for managing complex scenarios in remote sensing imagery [54].

Region-based Improvements: Incorporating Region Proposal Networks (RPN) within models like Faster R-CNN speeds up the generation of high-quality candidate regions, effectively minimizing background noise interference [55]. Multi-scale feature fusion via FPN and precise segmentation with Mask R-CNN addresses the challenge of detecting and classifying targets amidst complex backgrounds. Cascade R-CNN [56] and attention-guided region proposals further refine detection precision, especially for small targets, by incrementally purifying candidate regions and focusing more on significant image areas [57].

The advancements in network structure optimization for remote sensing image target detection not only enhance the performance of detection algorithms but also expand their capability to process complex scenes more effectively. As the field continues to evolve, future research is expected to introduce more innovative network architectures, further advancing the performance in remote sensing image target detection.

### 2.2. Optimization of One-Stage Detectors

Single-shot detectors, such as the You Only Look Once (YOLO) series, have garnered widespread attention in the field of remote sensing image target detection due to their real-time processing capabilities and relatively high detection accuracy [58]. These models integrate feature extraction, object classification, and position regression into a unified network, accomplishing target detection within a single forward pass, thus significantly enhancing processing speed.

The core concept of YOLO is to treat the target detection task as a single regression problem, directly mapping from image pixels to bounding box coordinates and class probabilities [59]. This design allows YOLO to achieve rapid detection as it circumvents the candidate region proposal stage present in traditional detection methods. YOLO has a distinct advantage in detection speed, achieving real-time processing speeds essential for rapid-response applications in remote sensing imagery. As YOLO considers the entire image during prediction, it can understand the global context of the image to some extent, helping to reduce false positives. The original YOLO model had limitations in detecting small objects and in precise localization, prompting improvements in subsequent versions.

YOLOv2 [60], presented by Joseph Redmon and Ali Farhadi at CVPR 2017, includes enhancements to the original YOLO, maintaining the same speed while being more powerful and capable of detecting up to 9000 categories. YOLOv2 introduced the concept of anchors, using predefined sets of bounding box dimensions for predicting target locations, thus improving the model’s capability to detect objects of various sizes. YOLOv2 employed the Darknet-19 network structure, which, compared to the original YOLO’s network structure, has fewer convolutional layers but improves speed while maintaining high detection accuracy. The introduction of passthrough layers in YOLOv2 enables finer target localization using shallow features, particularly enhancing small target detection performance. YOLOv2 adopts multi-scale input during training, increasing the model’s adaptability to objects of different sizes.

YOLOv3 [61] improved the detection performance for small objects by introducing multi-scale prediction and the Darknet-53 feature extraction network. It enhances the model’s recognition capabilities for objects of various sizes by making predictions at three different scales, leveraging both deep and shallow features.

YOLOv4 [62], while maintaining high-speed detection performance, further enhanced the model’s feature extraction capability and ability to handle complex backgrounds by introducing structures such as the Spatial Pyramid Pooling (SPP) block, Path Aggregation Network (PANet), and CSPDarknet53. The SPP block significantly increases the receptive field, extracting richer contextual features, whereas PANet improves learning capability through more effective feature fusion.

YOLOv5 [63] enhanced the model’s adaptability and detection efficiency for diverse targets in remote sensing imagery by introducing adaptive anchor calculations and automatic learning strategies for adjusting network width and depth. Moreover, optimizations in its model structure have significantly improved training and inference speeds.

YOLOv6 [64] continues the tradition of the YOLO series in providing models of various sizes for industrial applications to meet diverse performance and speed requirements. Unlike YOLOv4 and YOLOv5, as well as other anchor-based detection methods, YOLOv6 introduces an anchor-free detector. This improvement is likely aimed at simplifying the model and enhancing the flexibility and accuracy of detection.

YOLOv7 [39] builds on the efficient detection foundation of the YOLO series, incorporating advanced training techniques and architectural improvements, such as employing Transformer encoders as feature extractors to enhance the model’s understanding of complex scenes in remote sensing imagery. Additionally, it optimizes loss functions and training strategies, improving the model’s accuracy and robustness in remote sensing image target detection.

YOLOv8 [65] represents the latest in the YOLO series of object detection models, renowned for its high speed and accuracy. This version maintains rapid processing while enhancing the model’s feature extraction capabilities and generalization through architectural optimization and multi-scale training strategies. YOLOv8 also introduces an improved loss function and adaptive anchor box techniques to enhance detection precision across a variety of sizes and shapes of targets. These characteristics make YOLOv8 particularly well-suited for real-time object detection tasks in areas such as traffic monitoring, industrial automation, security surveillance, unmanned aerial vehicle monitoring, and automated retail checkout systems.

YOLOv9 [66], developed by the original YOLOv7 team, introduces the concept of Programmable Gradient Information (PGI) to address the diverse requirements needed by deep networks to handle multiple objectives. PGI provides comprehensive input information for calculating objective functions for targeted tasks, thereby delivering reliable gradient information for updating network weights. Additionally, a new lightweight network architecture called the Gradient Efficient Layer Aggregation Network (GELAN) has been designed. The architecture of GELAN demonstrates superior results with PGI in lightweight models, showcasing its effectiveness and efficiency.

### 2.3. Solve Special Challenges in Remote Sensing Images

Addressing the unique challenges presented by remote sensing imagery necessitates innovative approaches in the design and implementation of target detection algorithms. The intrinsic complexities such as varying scales, rotations, and occlusions of objects within these images pose significant hurdles for conventional detection methods. To overcome these challenges, recent advancements in the field have focused on developing scale-invariant and rotation-invariant network architectures, alongside employing multi-scale feature fusion techniques. These innovations are pivotal in enhancing the detection capabilities of models, ensuring they are adept at recognizing targets under a wide array of conditions, thus significantly improving the precision and resilience of remote sensing image target detection.

Scale-Invariant and Rotation-Invariant Architectures. Scale invariance in network architecture ensures that the model retains its detection capability regardless of the target size within the image. This is particularly important in remote sensing, where the same type of target may appear vastly different in size due to the altitude of the sensor or the perspective from which the image was captured. Similarly, rotation invariance allows the model to correctly identify objects regardless of their orientation, an essential feature given the arbitrary positioning of objects in the natural landscape captured by remote sensors. Implementing these invariances directly into the network architecture involves sophisticated techniques such as automatic scale adjustment layers and rotation-invariant feature extractors, which adjust the processing of the image data to compensate for these variabilities.

Multi-Scale Feature Fusion Techniques. Multi-scale feature fusion is another critical advancement that addresses the scale variability issue by integrating features extracted at different layers of the network. Early layers capture fine details that are crucial for identifying small objects, while deeper layers extract more abstract semantic information valuable for recognizing larger objects and understanding the scene context. By fusing these multi-layer features, the model can leverage both detailed and high-level cues for improved target detection across various scales.

This technique not only enriches the model’s feature set but also enhances its adaptability to complex backgrounds and cluttered scenes common in remote sensing imagery. The fusion process involves sophisticated mechanisms like attention models that weigh the importance of features from different layers, ensuring that the most relevant information is utilized for making predictions.

## 3. Preparation

### 3.1. Dataset

For our forthcoming work, we have prepared two datasets, namely the TGRS-HRRSD-Dataset and the NWPU VHR-10 Dataset.

The TGRS-HRRSD-Dataset has been extensively utilized across various research and application scenarios, particularly in the development and evaluation of target detection algorithms for high-resolution remote sensing imagery. The HRRSD dataset focuses on high-resolution remote sensing images, aiming to provide sufficient data support for identifying and analyzing small targets on the ground. The dataset comprises a total of 21,761 images, encompassing 55,740 target instances across 13 different remote sensing image (RSI) target categories, as shown in Table 1.

The images in the NWPU VHR-10 dataset are collected from various high-resolution satellite platforms, created by researchers from the Northwestern Polytechnical University (NWPU), China. The NWPU VHR-10 dataset utilized in this paper includes 10 categories, as shown in Table 2.

Both datasets underscore the importance of high-resolution images in remote sensing target detection and image analysis while demonstrating the potential applications under diverse environments and conditions. The data source diversity and target detection capabilities under complex environments of the TGRS-HRRSD-Dataset, along with the category imbalance issue demonstrated by the NWPU VHR-10 dataset with its clear and focused target categories, are key factors to consider when using these datasets for model training and evaluation. Moreover, both datasets employ meticulous data partitioning strategies to enhance the model’s generalization ability and promote fair and consistent performance assessment.

Furthermore, to address the issue of extreme imbalance in the number of target samples across different categories present in the datasets, during dataset partitioning, we statistically analyze the sample volume of dataset categories, dividing the dataset into training, validation, and test sets. The dataset partitioning strategy includes counting the number of samples in each category and striving to maintain a balanced data volume for each category during the partitioning of training, validation, and test sets.

### 3.2. Data Enhancement Processing

Target detection algorithms employ a variety of data augmentation methods to enhance the model’s generalization ability and performance, such as random cropping and scaling, random rotation and flipping, color jittering, crop padding, Mosaic data augmentation, MixUp data augmentation, and Perspective Transformation. These data augmentation techniques can be utilized individually or in combination to further improve the model’s adaptability to complex environments. YOLOv7 enhances the capability to detect targets across various scenes through these methods, especially in scenarios involving occlusions, varying sizes, and different background conditions.

In the context of processing large-scale remote sensing images, the input images are typically resized to a uniform dimension of 640 × 640. However, due to the memory limitations of deep learning models, it often becomes necessary to segment these images into smaller tiles (e.g., 1280 × 1280, 4096 × 4096, and 512 × 512). This segmentation strategy can lead to objects being divided and appearing across multiple sub-images, which in turn may result in their repeated detection and counting. To address these challenges, several strategies can be employed. First, implementing an overlap strategy, where an overlapping region is introduced between adjacent tiles to ensure that objects are fully visible in multiple contexts; second, developing cross-tile object tracking algorithms to identify and eliminate duplicate objects; third, utilizing advanced deep learning models that explicitly handle spatial coherence and object continuity, such as Graph Neural Networks; fourth, applying clustering or merging algorithms in the post-processing stage to integrate detections from various tiles; and lastly, employing edge-aware segmentation techniques to avoid slicing through critical objects. These methods not only enhance the accuracy of remote sensing image analysis but also significantly impact fields such as environmental monitoring, urban planning, and disaster management, thereby greatly improving the reliability and precision of decision-making processes.

To evaluate the performance of our enhanced algorithm in handling environmental variability, we employed data augmentation techniques to apply a series of transformations to the original images, thereby enriching the diversity of our dataset. This not only aids in simulating various environmental conditions but also enhances the model’s generalization capabilities. Specifically, we randomly selected images and adjusted their brightness and contrast to simulate different lighting conditions. Random noise was introduced to mimic sensor noise or adverse lighting conditions that may occur during image capture. We also manipulated the images by rotating and scaling to simulate observing targets from various angles and distances. Additionally, we incorporated effects such as raindrops, fog, and snow to mimic different weather conditions. These measures have strengthened the adaptability and robustness of the algorithm under varying environmental conditions.

**Figure 1:**
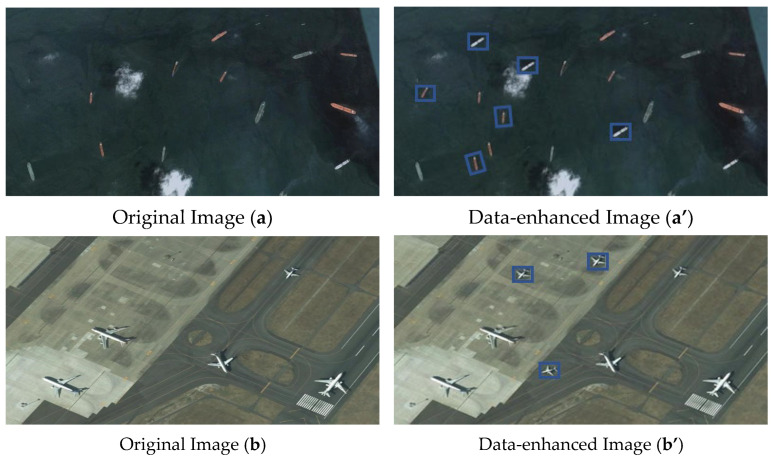
Data augmentation comparison chart. Includes original images and data-enhanced images.

In addition to the data augmentation methods adopted by YOLOv7, this paper also utilizes small target replication for data expansion, targeting the lesser number of small target samples in the dataset. Within an image, multiple small targets (based on the small target criteria defined by COCO) are randomly selected for replication and placed at random positions. Simultaneously, small objects are randomly replicated three times. This method helps increase the occurrence frequency of small targets, thereby improving the model’s ability to recognize these targets. The enhancement effect is illustrated in Figure 1. In order to clearly observe the data location of the additional items, the author added a blue box.

### 3.3. Implementation Setup

To carry out high-precision target detection tasks on remote sensing images, we employed the TGRS-HRRSD-Dataset and the NWPU VHR-10 Dataset. Our foundational system setup encompassed Ubuntu 18.04 as the operating system and PyTorch 1.11 as the deep learning framework, supported by CUDA version 11.3 and cuDNN version 8.2. The hardware configuration was powered by an NVIDIA RTX 3090 GPU, with 24GB VRAM, and an Intel(R) Xeon(R) Gold 6330 CPU, offering 56 virtual cores, to ensure substantial computational capabilities for our tasks.

For the training configuration, we utilized the Adam optimizer with a batch size of 28. The TGRS-HRRSD-Dataset underwent 500 training epochs to thoroughly comprehend the high-resolution details and complexities of the encompassed remote sensing images. In contrast, the NWPU VHR-10 Dataset was subjected to a relatively shorter training duration of 100 epochs, reflecting its specific characteristics and experimental requirements. This experimental setup was meticulously designed to enhance our novel model’s performance in detecting objects with high precision across different remote sensing image datasets, showcasing the model’s proficiency in managing various complex environmental conditions.

## 4. Three Novel Approaches

This paper first proposes a target detection method based on an improved YOLOv7 algorithm, incorporating a Multi-scale Feature Enhancement (MFE) module and an optimized anchor box generation process aimed at enhancing the detection performance of small targets in remote sensing images. By adjusting and enhancing the backbone network’s structure and optimizing the anchor box settings through data mining methods, this approach demonstrates significant advantages in improving the detection accuracy of small targets and reducing omissions. The method not only showcases innovative design concepts and implementation details but also highlights its potential application in the field of remote sensing image target detection through theoretical analysis and design rationale.

Furthermore, the paper elaborates on a target detection method involving a global information processing module, Depth information fusion Multilayer Perceptron (DP-MLP), integrated into the modified YOLOv7 structure. The core of this method addresses the issue of inadequate long-range dependency and global information capture in deep convolutional networks for target detection tasks, thereby enhancing model recognition accuracy and efficiency in complex environments. By incorporating the DP-MLP module into the improved YOLOv7 architecture, a novel target detection method is presented. This approach significantly boosts the accuracy of target detection and the model’s generalization ability through the fusion of deep and shallow features and optimized global information processing. The design of the DP-MLP module considers computational efficiency and improves the detection performance of small targets in complex scenarios like remote sensing images, providing new perspectives and solutions for the development of target detection technology.

Lastly, the paper delves into enhancing the performance of target detection models through a Semi-Supervised Learning Model (SSLM) approach that combines labeled and unlabeled data. The core of this section lies in leveraging the vast amount of available unlabeled data through generative models and pseudo-labeling strategies, thereby reducing reliance on extensive labeled data, effectively lowering data annotation costs, and enhancing the model’s generalization ability. By employing semi-supervised learning methods, this section presents an effective strategy for utilizing limited labeled data and a significant amount of unlabeled data to enhance target detection performance. With the application of CycleGAN-generated unlabeled data and pseudo-labeling strategies, the proposed method not only alleviates the burden of data annotation but also significantly enhances the model’s capability to handle complex visual tasks. This strategy offers a new solution pathway for target detection research in scenarios where data are scarce but task demands are high, promising broad applicational prospects.

### 4.1. Multi-Scale Feature Enhancement

This chapter aims to explore a multi-scale feature enhancement target detection method based on an improved YOLOv7 model. We aim to improve the model’s capacity to detect targets of various sizes by conducting a thorough analysis and improving the YOLOv7 backbone network, as well as introducing innovations in multi-scale feature extraction and fusion. Particularly, we aim to contribute to improving the detection accuracy of small objects and the robustness of the model in complex environments.

#### 4.1.1. Multi-Scale Enhancement Module Design for Backbone Network

In the field of target detection, accuracy and real-time performance are two crucial indicators of a model’s quality. The YOLO series, as a typical example of single-stage target detection methods, has garnered widespread attention for its efficient detection speed and commendable accuracy in real-time target detection applications. Particularly, YOLOv7, as the latest advancement in this series, has further enhanced detection performance through the introduction of several innovative technologies, including but not limited to multi-branch stacked structures, innovative subsampling mechanisms, special SPP (Spatial Pyramid Pooling) structures, and adaptive multiple positive sample matching. However, as application scenarios become more diverse and complex, the challenges faced by target detection are increasingly multiplying. Especially in the aspect of multi-scale target detection, traditional YOLOv7, despite achieving notable results, still exhibits some limitations. These limitations are primarily reflected in the lower detection accuracy for small objects and the need for improved target recognition capability in complex backgrounds. In light of these issues, a multi-scale feature enhancement method based on the improved YOLOv7 model appears particularly critical.

The backbone network of YOLOv7 is the most critical part of its architecture, tasked with extracting useful features from input images to lay the foundation for subsequent target detection tasks. During the feature extraction process, large-scale shallow feature maps contain a wealth of detailed information, making the classification and localization of small objects particularly important. Conversely, small-scale deep feature maps contain rich semantic information, ensuring the recognition of larger objects. If multi-scale feature fusion is employed to integrate the semantic information of shallow and deep features, the result is not only an abundance of complex classification features but also an enhanced perceptual ability of the target detection model toward small objects, thereby increasing the accuracy of detecting small targets. Additionally, multi-scale feature fusion can increase the size of the feature maps, enhancing the density of the prior boxes and thus avoiding the missed detection of small objects.

**Figure 2:**
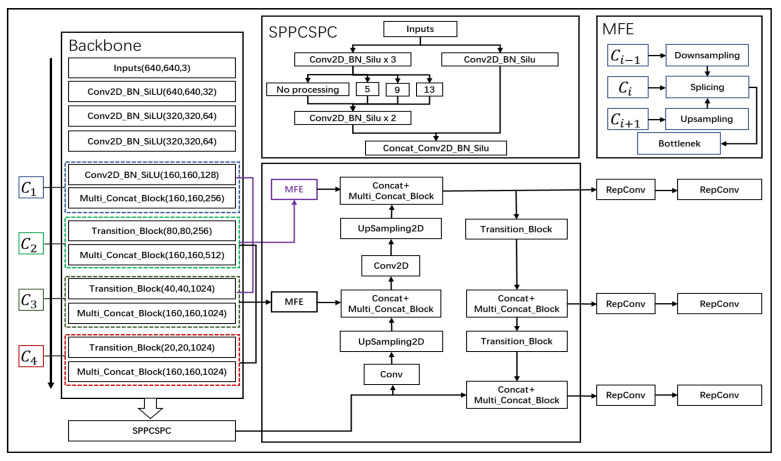
Multi-scale feature enhancement network structure diagram.

C1, C2, C3, and C4 are four modules divided according to the original backbone network, with their features extracted and then subjected to multi-scale feature fusion on the basis of the original network. By inputting the features from the C1, C2, and C3 modules into our designed MFE module, they are fused into a new feature. Similarly, the features from the C2, C3, and C4 modules are also fused into another new feature. The working principle of the MFE module involves the use of max pooling for downsampling and nearest-neighbor interpolation for upsampling. The detailed network structure is illustrated in Figure 2.

By fusing the features of four modules into two new features, the semantic information of shallow and deep features is integrated, leveraging the complementary advantages of both feature types to enhance model performance. Combining the detailed texture information from shallow features with the high-level context from deep features allows the model to detect objects of various scales and complexities more accurately, thereby improving the accuracy of target detection. The fusion strategy enriches the model’s feature representation by integrating different types of information. Such comprehensive feature maps are more robust, capable of handling a wide range of visual phenomena, making the model more versatile and effective across different tasks and datasets and enhancing the representation of features. Deep features provide context that can aid in the inference of partially occluded or cluttered objects’ presence, while shallow features capture the visible details, enhancing robustness against occlusion and clutter. The combination of local and global information offers a more holistic understanding of image content, helping the model generalize better then new unseen images. Additionally, this approach aids in improved segmentation and localization, as well as efficiency in learning and inference. Importantly, effectively implementing feature fusion does not significantly increase computational costs.

Among them, the maximum pooling Formula (1) is:

(1) $$P_{ij}=max(X_{2i,2j},X_{2i+1,2j},X_{2i,2j+1},X_{2i+1,2j+1})$$

where $P_{ij}$ is the information of the 80 × 0 or 40 × 40 feature map and $X_{m,n}$ is the information of the original 160 × 160 or 80 × 80 feature map.

This article uses the nearest neighbor interpolation algorithm to map each original pixel to a 2 × 2 area (80/40 = 2, 40/20 = 2). For each pixel, $P^{\prime }\left(i^{\prime },j^{\prime }\right)$ is obtained according to the pixel P(i,j) in the corresponding source image and its Formulas (2) and (3) is

(2) $$i=round\left(\frac{i^{\prime }}{2}\right),j=round\left(\frac{j^{\prime }}{2}\right)$$

(3) $$P^{\prime }\left(i^{\prime },j^{\prime }\right)=P(i,j)$$

Among them, i, j, $i^{\prime }$, and $j^{\prime }$ represent positions, that is, coordinates; round represents the round function used for rounding.

#### 4.1.2. Optimizer of Anchor Box Generation

In target detection tasks, the design of anchor boxes plays a crucial role in the performance of the model. Anchor boxes are predefined sets of fixed-size rectangular boxes that the target detection model uses as references to predict the positions and categories of actual targets. Traditionally, the sizes of these anchor boxes are manually set, possibly based on experience or rough analysis of a specific dataset. However, this approach may not provide the optimal anchor box sizes for specific tasks, especially when the size distribution of targets in the dataset is highly diverse.

K-means clustering is a widely used clustering algorithm that partitions the data into k clusters by minimizing the sum of squared distances from each point to its assigned cluster center. In the context of determining anchor box sizes, the input to the algorithm is the widths and heights of all target bounding boxes in the dataset, with the objective to find k cluster centers that represent the optimal anchor box sizes.

**Figure 3:**
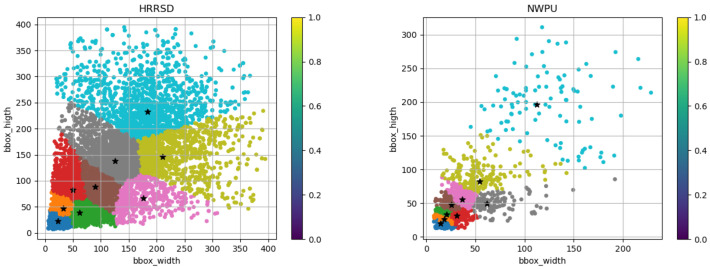
K-means clustering with Larger k; TGRS-HRRSD-Dataset is on the left and NWPU VHR-10 Dataset is on the right. In the image, each target frame is classified to the nearest cluster center, marked with a different color, and the cluster center is marked with a large asterisk. This can visually display the clustering of target boxes.

The target objects in the dataset of this paper deviate in size from those typically found in natural scenes. Utilizing anchor box scales derived from natural images would generate a large number of superfluous redundant anchor boxes, thereby wasting substantial computational resources and extending training time. To ensure the model’s anchor boxes more closely match the size of the target bounding boxes in our dataset, we employ the K-means clustering algorithm to cluster the target bounding boxes and determine the optimal anchor points. This process is tantamount to an in-depth data mining of the dataset, clustering the target boxes of similar scales. The final anchor box positions are illustrated in Figure 3.

#### 4.1.3. Loss Function

The loss function in this article mainly consists of three parts, including positioning loss, classification loss, and confidence loss. The localization loss is used to measure the position difference between the predicted box and the real box; the classification loss is used to measure the prediction accuracy of the target category in the predicted box and the confidence loss is used to express the model’s confidence in whether each prediction box contains the target.

If the prediction box correctly contains the object, the prediction confidence should be 1, otherwise it is 0. The purpose of the confidence loss is to train the model to increase the confidence in predictions that contain the target, while decreasing the confidence in predictions that do not contain the target. Its formula is expressed as (4)

(4) $$L=L_{\_coord}+L_{\_conf}+L\_cls$$

Among them, $L_{\_coord}$ represents the positioning loss, $L_{\_conf}$ represents the confidence loss, L_cls classification loss uses BCELoss (5) for target confidence loss and classification loss, and CIoU loss (6–8) is used for positioning loss.

(5) $$Loss=-\frac{1}{N}\sum_{i=1}^{N}\left[y_{i}\log\left(p_{i}\right)+(1-y_{i})\log(1-y_{i})\right]$$

(6) $$V=\frac{4}{\pi^{2}}{\left(arctan\frac{w^{gt}}{h^{gt}}-arctan\frac{w}{h}\right)}^{2}$$

(7) $$\alpha =\left\{\begin{array}{c} 0,IoU<0.5\\ \frac{V}{\left(1-IoU\right)+V^{\prime }},IoU\geq 0.5 \end{array}\right.$$

(8) $$L_{CIoU}=1-IoU+\frac{\rho^{2}\left(P,P^{gt}\right)}{c^{2}}+\alpha V$$

In the formula, $w^{gt}$ and $h^{gt}$ represent the width and height of the real frame, w and h represent the width and height of the predicted frame, V is used to detect whether the aspect ratio of the two is the same, α is the balance parameter, and P is the predicted frame. $P^{gt}$ is the real box. When V=0, $L_{CIoU}$ cannot be expressed stably. The CIOU loss function takes into account the overlapping area, center point distance, and aspect ratio of bounding box regression.

### 4.2. MLP Module Design of Global Information

**Figure 4:**
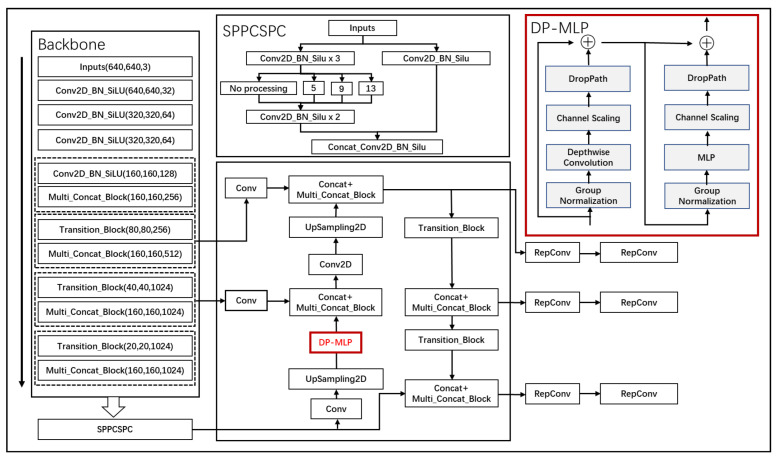
DP-MLP structure diagram.

Deep convolutional backbone networks are making impressive progress in areas such as image classification, target detection, and instance segmentation. While 3 × 3 convolutional kernels are utilized in these backbones to capture local information effectively, it is crucial to model long-range dependencies in visual tasks like target detection. The recognition of objects in such tasks often necessitates consideration of the relationship between the target and its surrounding context. Understanding the entire image context and background is vital; global information allows the model to incorporate insights from the whole image, providing a more comprehensive contextual understanding beyond just local areas. This enhances the model’s ability to discern the relationships between objects and their environment, thereby improving the accuracy of object recognition. To address this, our work introduces a novel architecture that blends local feature extraction with a design incorporating MLP modules capable of capturing long-range information, termed DP-MLP, as shown in Figure 4.

DP-MLP represents a tailored architecture that integrates local feature processing with the ability to perceive and assimilate extended spatial relationships within an image. This is achieved by partitioning the feature extraction process into distinct phases, where initially, local patterns are identified using smaller convolutional kernels, such as the traditional 3 × 3 convolutions that excel in capturing detailed textural and shape information.

Subsequently, the architecture transitions to leverage MLP modules, which are specifically designed to handle the long-distance information that is pivotal for comprehending the broader context of an image. These modules operate on the principle of processing information across the entire spatial extent of the input, thus enabling the network to understand and integrate global contextual cues that are essential for accurate target detection.

The DP-MLP module consists of two sub-modules and each sub-module contains a series of different components. The first sub-module mainly focuses on depth-separable convolution and correlation processing, while the second sub-module is more focused on the application of MLP. These two modules are fused with the input feature map through residual connections. For the input feature map X, the operation of the first sub-module can be expressed as (9)

(9) $$Y_{1}=x+DP(CS\left(DWConv\left(GN\left(x\right)\right)\right))$$

The operation of the second submodule can be expressed as (10)

(10) $$Y_{2}=x+DP(CS\left(DWConv\left(GN\left(Y_{1}\right)\right)\right))$$

Finally, it is fused with the feature map of the first module through residual connection. The final feature map after passing through the DP-MLP module is expressed as (11)

(11) $$Y=Y_{1}+Y_{2}$$

In the above formula, *X* is the input. *GN* stands for Group Normalization operation. *DWConv* stands for Depthwise Convolution operation. *CS* stands for Channel Shuffle operation. *MLP* stands for Multilayer Perceptron operation. *DP* stands for Dropout operation. *Y*_1_ and *Y*_2_ are outputs. *Y* is the final output.

DropPath is a regularization technique used to reduce overfitting and improve the generalization ability of deep neural networks. It does this by randomly discarding (i.e., not updating) a portion of the paths in the network during training, as shown in Equation (12):

*x* = *x* + *DropPath(conv(x))* (12)

The DP-MLP approach ensures that while the model remains sensitive to the nuances of local features, it also develops a holistic view of the image, effectively bridging the gap between detailed local analysis and global contextual understanding. This dual-focus strategy significantly enhances the model’s capacity to recognize and classify objects within complex scenes where both local details and the wider scene context contribute to accurate identification and segmentation.

### 4.3. Semi-Supervised Model for Unlabeled Data

In the realm of deep learning, the role of data is pivotal in molding the performance of models and in driving the progress of research. The significance of data stems from its integral role in training models, profoundly impacting their generalization ability, degree of fit, and learning prowess. Yet, in many domains, while there is an abundance of unlabeled data readily available, the acquisition of labeled samples often requires specialized apparatus or entails an expensive and lengthy manual annotation process. In order to resolve this difference, this chapter explores the method of semi-supervised learning to train target detection models using limited labeled data and large amounts of unlabeled data.

This research initiative leverages the prowess of semi-supervised learning, employing a strategic combination of limited annotated data and a large volume of unannotated data to train an target detection model. The CycleGAN model, a variant of Generative Adversarial Networks (GANs), serves as the cornerstone for generating an additional unannotated dataset. It achieves domain adaptation by adopting adversarial training, aligning the distribution of generated images with those of the target domain to accomplish image-to-image translation. The inclusion of a cycle consistency loss ensures the translation maintains consistency between domains, producing images of a more realistic quality.

**Figure 5:**
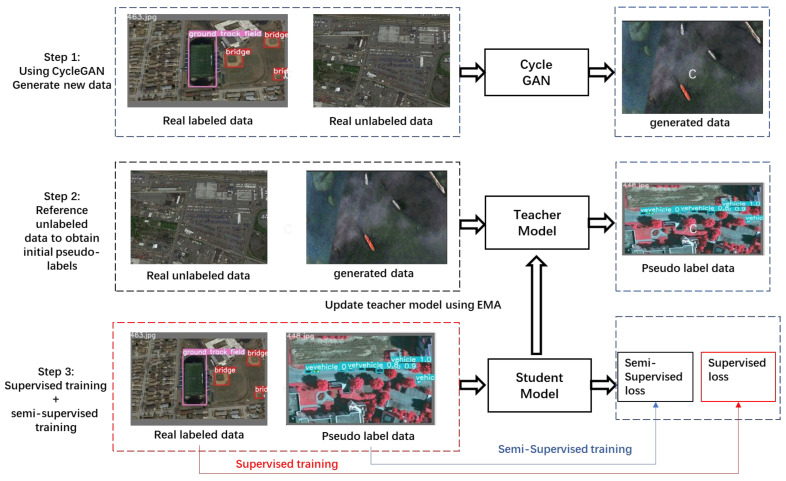
Semi-supervised model structure diagram.

Simultaneously, a pseudo-label strategy, commonplace in semi-supervised learning paradigms, is employed. This involves utilizing pseudo-labels generated by a teacher model on unannotated data to supplement the training of a student model. Such an approach maximizes the utility of the unannotated data, enhancing the model’s performance while mitigating reliance on extensive annotated datasets, thereby effectively alleviating the costs and complexities associated with data annotation. The frame structure is shown in Figure 5.

The NWPU VHR-10 Dataset was subjected to this methodology. The entire dataset was processed through the CycleGAN model, which served dual purposes, first, to expand the dataset, and second, to enable the model to learn invariant features across domains. This generated a new unannotated dataset that was used in conjunction with the semi-supervised learning model. The data distribution for training the CycleGAN model included a training set of 650 images, which subsequently led to the generation of an additional 520 unannotated images. These, along with the original 150 unannotated images provided by NWPU VHR-10, formed the dataset for semi-supervised training. Consequently, the data split for the semi-supervised model training was as follows: 312 images for training, 208 images for validation, and 130 images for testing. An additional set of 670 images was leveraged to generate pseudo-labels using the teacher model.

The relationship between the teacher model and the student model: The weight of the student model updates the weight of the teacher model through EMA (Exponential Moving Average). EMA weight update refers to the “Exponential Moving Average” weight update.

The working principle of EMA weight update is that at each training step, the weight of the teacher model will be updated based on the weight of the student model. The update rules are as follows in Equation (13):

(13) $$W_{tea}=\alpha W_{tea}+\left(1-\alpha \right)\;W_{stu}$$

α is a number close to 1 and this article uses 0.999, which means that the weight of the teacher model largely retains the previous value while slightly integrating the current weight of the student model. This method can smooth the update of model parameters and reduce fluctuations during the training process, thereby improving the model’s generalization ability on unseen data.

The semi-supervised overall loss function and supervised loss function in this article as Equations (14) and (15), as follows:

(14) $$L_{total}=L_{sup}+L_{unsup}$$

(15) $$L_{unsup}=\frac{1}{N_{cls}}\sum_{i=1}^{N_{cls}}L_{cls}(s_{i},{s\prime }_{i})+\frac{1}{N_{loc}}\sum_{i=1}^{N_{loc}}L_{loc}\left(b_{i},{b\prime }_{i}\right)+\frac{1}{N_{loc}}\sum_{i=1}^{N_{loc}}L_{iou}\left(p_{i},{p\prime }_{i}\right)$$

Among them, $N_{cls}$ and $N_{loc}$ are the number of samples for classification and positioning, respectively, $L_{cls}$ and $L_{iou}$ are defined in Equations (16) and (17), and $L_{loc}$ represents the CIoU loss.

(16) $$L_{cls}=FL\;(S‘,S)$$

(17) $$L_{iou}=BCE({S‘}_{iou},IOU)$$

where FL represents FocalLoss and BCE represents the cross-entropy loss function. The letters with ‘ in the above formula are the results of network prediction. The supervised loss is the same as the loss function of target detection yolov7.

## 5. Experimental Results

To clearly and intuitively present the experimental results of the three innovative methods we propose, we initially conducted comparisons of each method against alternative approaches, followed by a comprehensive comparison and analysis of the overall effects of the three methods. Overall, our methods have achieved commendable performance. The specific analyses are detailed as follows.

### 5.1. Multi-Scale Feature Enhancement Experimental Results

This section presents the experimental results obtained from the implementation of the MFE method within our target detection framework. The MFE method aims to integrate features from different levels of the network to improve the detection accuracy, particularly for small and medium-sized objects, as shown in Table 3 and Table 4.

In the experimental comparison conducted on the TGRS-HRRSD-Dataset and NWPU VHR-10 Datasets, the target detection algorithms were evaluated based on their Average Precision (AP) for various classes and their overall Mean Average Precision (MAP). Notably, the Ours algorithm consistently outperformed other state-of-the-art methods across most classes and achieved the highest MAP values of 93.4% and 93.1% on the TGRS-HRRSD-Dataset and NWPU VHR-10 Datasets, respectively. This suggests that the features and techniques employed by the Ours algorithm are more effective for the given datasets. It is also observed that certain algorithms, such as Fast R-CNN, displayed substantial variation in performance across different classes, indicating potential overfitting to specific class features or underfitting to others. The performance disparity among the algorithms on different datasets highlights the influence of dataset characteristics on algorithm efficacy. Further analysis of the specific attributes of the datasets and the design of the algorithms would be essential to understand the underlying factors contributing to this performance variation.

**Figure 6:**
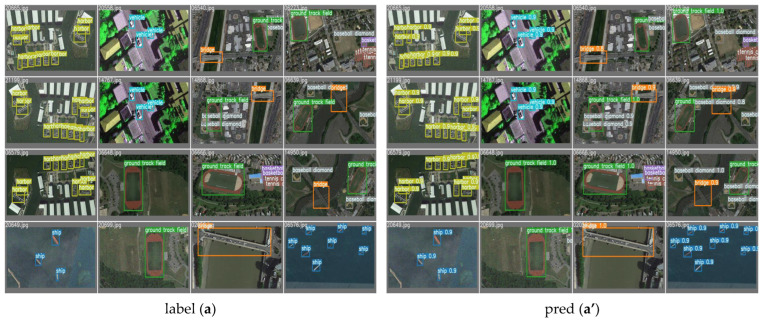

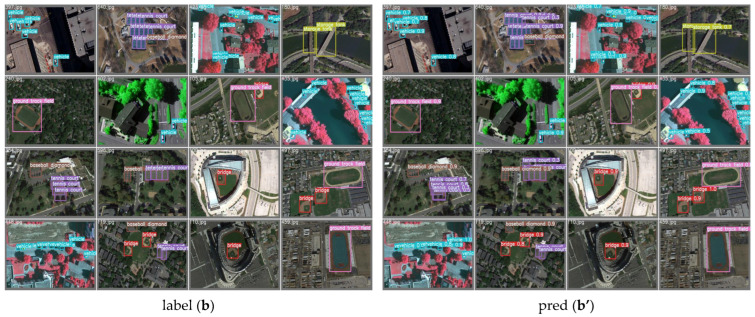
Visualization renderings. Picture “**a**” is the TGRS-HRRSD-Dataset data and Picture “**b**” is the NWPU VHR-10 Dataset data.

To effectively demonstrate the exceptional performance of our model, we have presented visualization charts depicting the model’s predictions. The images display the correct labels for each target alongside the outcomes predicted by our model. The results show a close overlap between the predicted bounding boxes and the actual annotated boxes, indicating our model’s proficiency in accurately locating targets. The predicted category labels are consistent with the true labels, underscoring our model’s high classification accuracy. Moreover, these visualizations allow for an intuitive assessment of the model’s precision in identifying and positioning various objects, such as airplanes, ships, racetracks, and parking lots, including potential instances of missed detections, false positives, or inaccuracies in localization, as shown in Figure 6.

### 5.2. MLP Module Design of Global Information Experimental Results

In this work, depthwise separable convolutions are amalgamated with MLP to enhance the efficiency and performance of the network. The role of depthwise separable convolutions is to reduce computational load and the number of parameters, thereby augmenting the network’s efficiency while concurrently enriching its capacity to comprehend global information from the input. To counteract the performance degradation associated with increased network depth, a DropPath regularization is integrated into the deep network architecture, which is instrumental in capturing long-range dependencies. This inclusion serves to optimize the model for superior performance. The experimental results are shown in Table 5 and Table 6.

In this comparative study, we evaluate the performance of the DP-MLP model against established target detection algorithms across two challenging remote sensing datasets: the TGRS-HRRSD-Dataset and NWPU VHR-10 Dataset. The empirical results elucidate the DP-MLP’s superior ability to discern and localize objects in high-resolution aerial imagery.

On the TGRS-HRRSD dataset, the DP-MLP model outperforms conventional methods such as Mask R-CNN, RS-YoloX, and even the more recent Yolo-v7, across a spectrum of target categories. Specifically, the DP-MLP achieves an impressive 93.1% MAP, a substantial improvement over the next best-performing method, Yolo-v7, which attains a 92.9% MAP. Such advancement is particularly pronounced in the detection of ‘ship’ (95.7%), ‘storage tank’ (97.5%), and ‘vehicle’ (97.8%), underscoring the DP-MLP’s efficacy in processing global and local contextual cues pivotal for these categories.

Furthermore, when assessed on the NWPU VHR-10 Dataset, the DP-MLP continues to demonstrate its dominance, with a 92.1% MAP that stands well above the 91.2% achieved by the competing Yolo-v7. Remarkably, in the ‘airplane’ category, the DP-MLP model shows a leap in performance to 99.5%. In the “Ship” category, the performance of the DP-MLP model jumps to 77.3%. The detection effects of both small target images reached the best among all models.

The performance superiority of the DP-MLP model can be attributed to its innovative architectural design, which harmonizes the depthwise separable convolutions with MLPs capable of effectively capturing long-range dependencies. This synergy facilitates a comprehensive understanding of the scene at large, allowing for intricate object interactions and environmental relationships to be factored into the detection process, a critical requirement in the domain of remote sensing.

**Figure 7:**
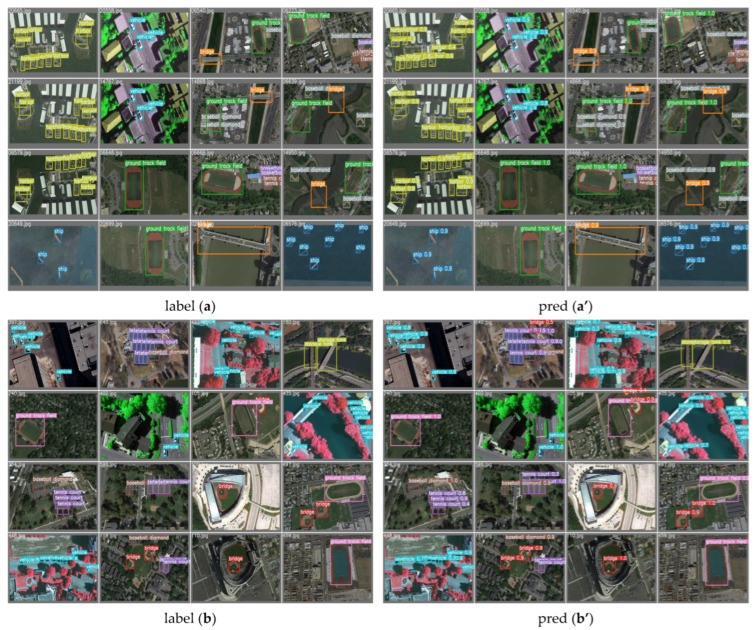
Visualization rendering. Picture “**a**” is the TGRS-HRRSD-Dataset data and Picture “**b**” is the NWPU VHR-10 Dataset data.

Visualization of DP-MLP is conducted for enhanced target detection in remote sensing imagery. Through these visualizations, we offer a transparent and detailed examination of the DP-MLP’s performance, highlighting its superior detection capabilities over conventional methods. The high fidelity of the model’s predictions to the actual objects within the images serves as a testament to the robustness and accuracy of the DP-MLP architecture, validating its application for advanced remote sensing tasks, as shown in Figure 7.

### 5.3. Semi-Supervised Model for Unlabeled Data Experimental Results

**Figure 8:**
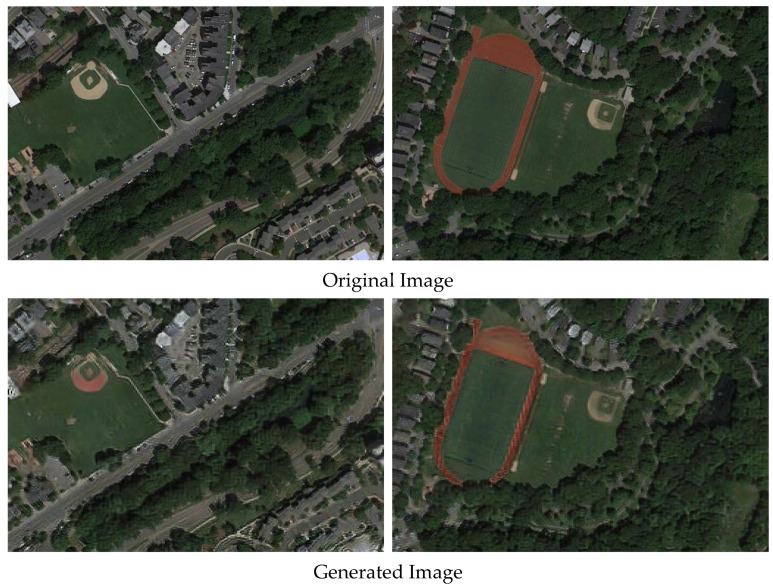
Comparison of the original image and generated image.

This semi-supervised approach, underpinned by CycleGAN-generated datasets, promises a significant advancement in target detection by effectively utilizing synthetic images to train robust models capable of higher levels of abstraction and generalization. The comparison between the original image and the generated image is shown in Figure 8. The experimental results are shown in Table 7.

The comparative experimental results elucidate the efficacy of our model in target detection tasks. Performance metrics are gauged by the Intersection Over Union (IOU) and Average Precision (AP) across various models. The model we propose achieves a notable AP of 91.2% at an IOU threshold of 0.50 and sustains an impressive 62.4% AP even under the stringent IOU range of 0.50:0.95, a testament to its superior performance within the industry. In contrast, the ARSL model scores 80.4% and 48.4% for the respective metrics, while the YOLOv7 model exhibits a close 90.7% and 61.4%.

The data unequivocally indicates that our model surpasses the ARSL model at both IOU thresholds and edges out YOLOv7 at the higher IOU = 0.50:0.95 standard. This emphasizes the superior accuracy of our model, particularly its robustness and generalizability in complex scenarios. The results validate the effectiveness of the semi-supervised learning strategy in enhancing the performance of target detection models, especially when annotated data are scarce. This methodology not only boosts model performance but also mitigates the reliance on extensive annotated datasets, presenting a cost-effective solution.

**Figure 9:**
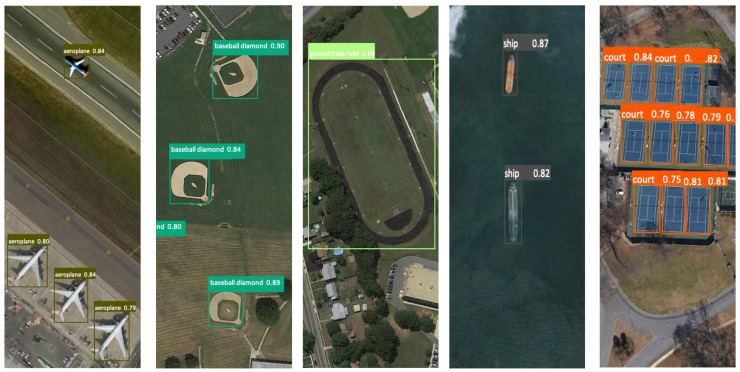
Visual display of detection results.

The provided visualizations serve as an empirical testament to the prowess of the implemented semi-supervised target detection model. The model’s acuity in identifying and classifying a variety of objects within aerial imagery is corroborated by the exhibited confidence scores. Exemplified by the detection of airplanes positioned on the tarmac, baseball diamonds etched within verdant fields, ships navigating maritime expanses, and tennis courts and ground track fields integrated within urban environs, the model demonstrates an astute precision in labeling distinct entities, as shown in Figure 9.

These depictions affirm the model’s resilience, illustrating its capacity to harness unlabeled data to fortify and refine its detection algorithms effectively. The incorporation of pseudo-labels emanating from the model’s inference on unlabeled data significantly augment the model’s learning apparatus, empowering it to ascertain with high certainty across a multiplicity of terrains and objects. Delving into the depths of the visual corpus, one observes a model that not only performs with exemplary competence but also epitomizes the transformative potential of semi-supervised learning paradigms within the ambit of target detection.

### 5.4. Comparison of Overall Experimental Results

This section compares the effects of three innovative methods for target detection in remote sensing imagery, utilizing two distinct datasets. The methods analyzed include MFE, the DP-MLP, and SSLM. Below is a comparative analysis of the performance of these methods on the TGRS-HRRSD-Dataset and NWPU VHR-10 Datasets, as shown in Table 8 and Table 9.

MFE: This approach, through improvements to the YOLOv7 model, has bolstered the model’s ability to detect targets of various scales, particularly small targets. Experimental results on the TGRS-HRRSD and NWPU VHR-10 datasets demonstrate superior performance in several categories, with MAP values reaching 93.4% and 93.1%, respectively.

DP-MLP: The DP-MLP module enhances target detection models by blending local feature extraction with global information processing. This method specifically focuses on how the model uses the overall image context to enhance recognition accuracy. Testing on the two datasets shows that DP-MLP outperforms YOLOv7 and other benchmark models across most target categories, achieving a MAP of 93.1% on the TGRS-HRRSD dataset and 92.1% on the NWPU VHR-10 dataset.

SSLM: This model addresses the challenge of limited labeled data and abundant unlabeled data by employing Generative Adversarial Networks (CycleGAN) and pseudo-labeling strategies to expand the training dataset. This strategy significantly reduces data annotation costs while enhancing the model’s generalization capabilities. Experimental results indicate that this method performs exceptionally well under standard IOU metrics, notably surpassing YOLOv7, YOLOv8, and other semi-supervised models.

It should be noted that although YOLOv7 showed slightly higher accuracy on the two categories “tennis court” and “basketball court”, this may be because these two categories usually belong to large targets. Therefore, the model may have strong capabilities in detecting large targets. The focus of this improvement is to improve the model’s perception of small targets, improve its ability to process complex backgrounds, and enhance the use of global information. These improvements can be achieved by adjusting the network structure and introducing more complex feature fusion methods, data enhancement techniques, and contextual information.

From Table 10, it is evident that the MFE, DP-MLP, and SSLM models outperform YOLOv7 and YOLOv8 in terms of accuracy, particularly highlighting their suitability for scenarios demanding high precision. Both MFE and DP-MLP demonstrate superior model performance and strong generalization capabilities with over 93% mAP on the TGRS-HRRSD and NWPU VHR-10 datasets. Although these models have higher resource consumption, especially in terms of FLOPs and parameter count, their high accuracy justifies the substantial resource investment. SSLM, while slightly less accurate, shows advantages in operational efficiency with its high FPS and lower resource demands, making it particularly well-suited for applications requiring rapid processing. Overall, these models exemplify the necessary resource and design trade-offs when pursuing extremely high recognition accuracy, making them apt for complex visual tasks where precision is paramount and resources are not the primary constraint.

**Figure 10:**
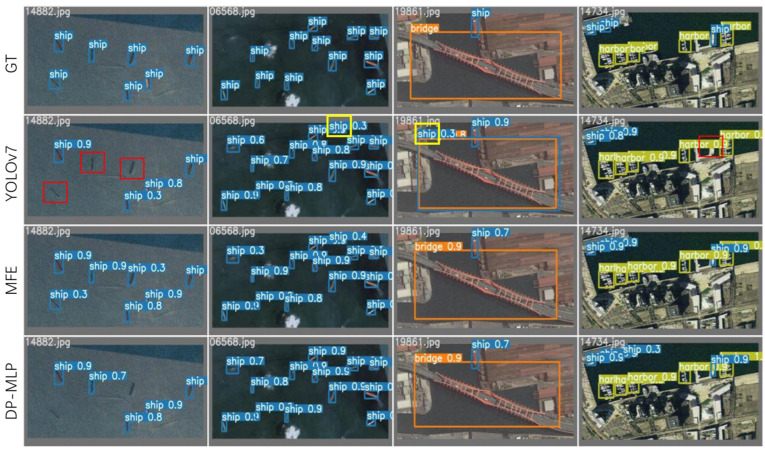
Comparison of detection performance based on the TGRS-HRRSD Dataset. In the figure, GT denotes ground truth labels, while YOLOv7/MFE/DP-MLP represents the three comparison models. The red boxes indicate missed detection annotations and the yellow boxes denote false detection annotations.

**Figure 11:**
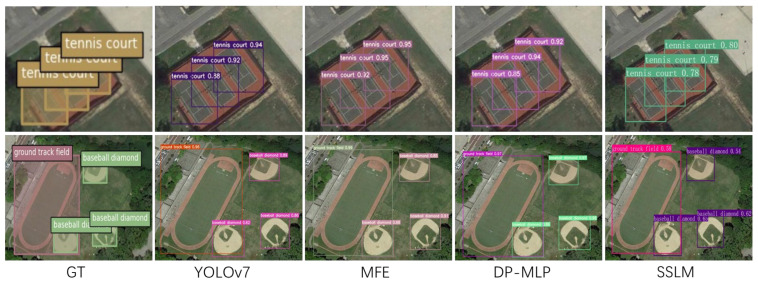
Comparison of detection performance based on the NWPU VHR-10 dataset. In the figure, GT denotes ground truth labels, while YOLOv7/MFE/DP-MLP/SSLM represents the three comparison models.

For a more intuitive comparison of the detection performance among various models, we assembled the visualized results of detection for comparison. Through this comparison, we observed instances of missed detections and false detections in the original YOLOv7 model. Our model demonstrates superior detection performance compared to the original YOLOv7 model. Additionally, our model exhibits superior detection accuracy. Regardless of the dataset used, target size, complexity of image backgrounds, presence of occlusions, pixel clarity, and other factors, our model consistently demonstrates superior performance. Refer to Figure 10 and Figure 11 for detailed illustrations.

**Figure 12:**
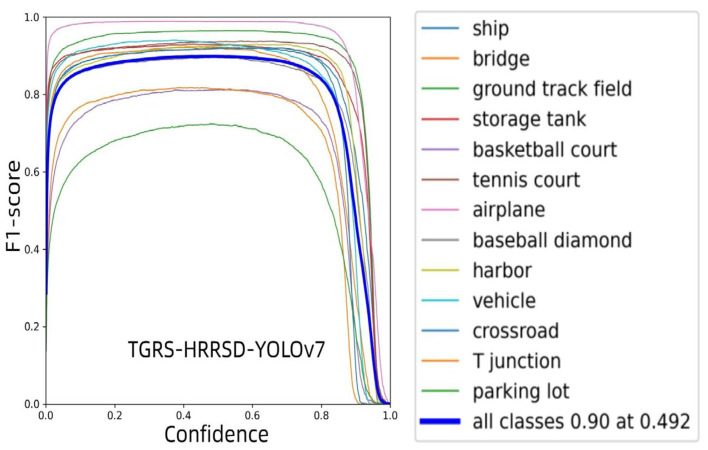

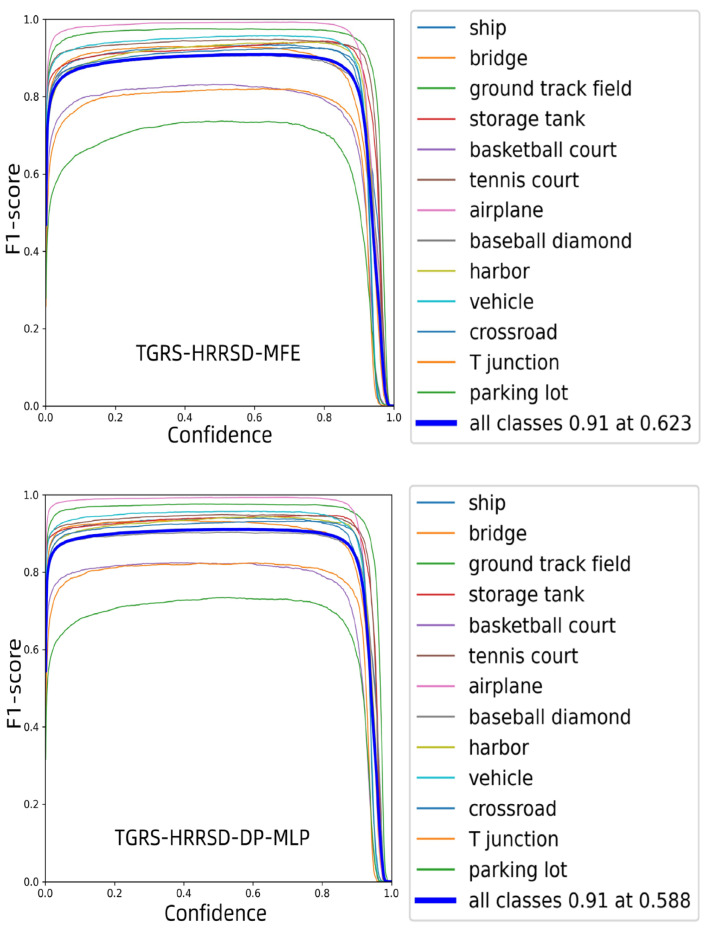
Comparison of F1 scores of YOLOv7, MFE, and DP-MLP on the TGRS-HRRSD data set.

Figure 12 shows the performance of three different models: YOLOv7, MFE, and DP-MLP in detecting different categories of targets in the TGRS-HRRSD-Dataset. The F1 score serves as a measure of model performance, taking into account both precision and recall. In these plots, the *x*-axis represents confidence, while the *y*-axis represents the F1 score.

The overall performance of YOLOv7 is the lowest among all models, with the F1 score reaching 0.9 only at higher confidence thresholds. The performance of the MFE model in different categories is relatively balanced and its overall performance is significantly higher than YOLOv7, especially at the lower confidence threshold. Its overall F1 score is about 0.91 and the confidence threshold to reach this standard is 0.623. The DP-MLP model shows a similar overall performance to MFE, with an overall F1 score of 0.91, but the confidence threshold for reaching this criterion is slightly higher at 0.588.

Advantages of MFE and DP-MLP models: MFE may enhance the model’s ability to understand complex scenes by integrating multiple feature sources. This is particularly evident in the detection of specific categories such as “ground runway” or “parking lot”, where the F1 scores are the highest among all models. This shows that the MFE model has advantages in handling diverse features.

DP-MLP appears to reduce reliance on confidence thresholds while maintaining high F1 scores. Although slightly higher than the MFE model at reaching the confidence threshold of 0.91 F1 score, it maintains high-performance stability across the entire confidence range.

In summary, the MFE model shows strong performance in handling diverse scene features, while the DP-MLP model shows advantages in overall stability. Both models have shown significant advantages over the traditional YOLOv7 model in improving performance under low confidence.

The comparison reveals distinct advantages for each method. The Multi-Scale Feature Enhancement method excels in handling small targets and complex scenes; the DP-MLP module, by enhancing global information processing, improves recognition accuracy; while the Semi-Supervised Learning Model effectively utilizes unlabeled data, reducing dependency on extensive labeled datasets and lowering costs while improving model usability. These innovative methods not only enhance the precision of target detection but also provide new directions and insights for research in remote sensing imagery target detection.

## 6. Conclusions

The research introduces a refined version of the YOLOv7 algorithm, accentuated by an enhanced multi-scale feature enhancement methodology, a novel YOLOv7 global information MLP module, and the integration of a semi-supervised target detection approach leveraging unlabeled data. The experimental results substantiate the method’s superior performance over the existing YOLOv7 framework across various metrics, demonstrating substantial improvements in detection accuracy, particularly in small target detection and in environments with complex target arrangements.

This study’s foremost contribution lies in its innovative enhancement of the YOLOv7 algorithm, which markedly improves target detection performance in remote sensing imagery. The introduction of a multi-scale feature enhancement technique and a global information MLP module represents a pioneering step in capturing and integrating both detailed and global information within images, thereby significantly bolstering target detection accuracy. Furthermore, the exploration of semi-supervised learning techniques utilizing unlabeled data to augment the model’s performance encapsulates a vital contribution, showcasing a cost-effective strategy for enhancing detection systems under constrained annotation resources.

Future research avenues should explore the integration of more advanced machine learning techniques, such as deep reinforcement learning and few-shot learning, to further refine the detection accuracy and efficiency. Additionally, the adaptability of the proposed enhancements in diverse remote sensing applications, ranging from urban planning to environmental monitoring, warrants rigorous examination. While the research achieves notable advancements, it acknowledges the potential limitations associated with the scalability of the semi-supervised learning model and the computational demands of the enhanced YOLOv7 algorithm, suggesting a balance between performance gains and computational efficiency as an area for further investigation.

In conclusion, this research not only propels the understanding and capabilities within the domain of remote sensing image target detection but also lays a foundational framework for future innovations in the field. Its contributions resonate through the improved accuracy and efficiency of target detection models, fostering new insights and methodologies that can be leveraged across a broad spectrum of remote sensing applications.

## Figures and Tables

**Table 1:** TGRS-HRRSD-Dataset data table. In this table, ‘Train’, ‘Val’, and ‘Test’ are three subsets of the dataset. M-R-S (Mean Resized Scale) shows the average scale of each category. R-S-S (Resized Scale Std) is the standard deviation of the category scale.

Label	Name	Train	Val	Trainval	Test	All	M-R-S/Pixel	R-S-S/Pixel
1	ship	950	948	1898	1988	3886	167.44	110.37
2	bridge	1123	1121	2244	2326	4570	246.1	110.53
3	ground track field	859	856	1717	2017	3734	276.5	100.65
4	storage tank	1099	1092	2191	2215	4406	125.6	68.41
5	basketball court	923	920	1843	2033	3876	108.19	57.46
6	tennis court	1043	1040	2083	2212	4295	102.71	38.8
7	airplane	1226	1222	2448	2451	4899	113.21	67.98
8	baseball diamond	1007	1004	2011	2022	4033	231.61	117.85
9	harbor	967	964	1931	1953	3884	163.96	94.16
10	vehicle	1188	1186	2374	2382	4756	41.96	9.99
11	crossroad	903	901	1804	2219	4023	220.54	59.24
12	T junction	1066	1065	2131	2289	4420	198.71	54.88
13	parking lot	1241	1237	2478	2480	4958	122.85	54.45

**Table 2:** NWPU VHR-10 Dataset table. In this table, ‘Train’, ‘Val’, and ‘Test’ are three subsets of the dataset.

Label	Name	Train	Val	Test	All	Size/Pixel
1	airplane	467	180	110	757	50 × 77–104 × 117
2	ship	145	62	95	302	20 × 40–30 × 52
3	storage tank	442	120	93	655	27 × 22–61 × 51
4	tennis court	255	107	162	524	45 × 54–122 × 127
5	basketball court	83	38	38	159	52 × 52–179 × 179
6	ground track field	91	28	44	163	195 × 152–344 × 307
7	harbor	149	55	20	224	95 × 32–175 × 50
8	bridge	95	15	14	124	88 × 90–370 × 401
9	vehicle	376	129	93	598	20 × 41–45 × 80
10	baseball diamond	242	59	89	390	66 × 70–109 × 129

**Table 3:** Comparative experimental results of MFE on the TGRS-HRRSD-Dataset. (Rate: %).

Label	Name	YOLOv2	Fast R-CNN	Faster R-CNN	YOLOv4	YOLO-RS	YOLOv7	MFE
1	ship	78.5	75.0	88.5	90.18	90.21	95.0	95.8
2	bridge	79.0	75.1	85.5	90.47	90.48	94.3	95.2
3	ground track field	94.4	90.0	90.6	90.52	90.69	98.9	99.1
4	storage tank	72.4	79.8	88.7	90.39	90.33	96.1	97.2
5	basketball court	62.2	83.6	86.9	69.44	77.52	83.1	85.1
6	tennis court	67.6	75.0	80.7	90.34	90.37	97.0	97.3
7	airplane	84.6	83.3	90.8	90.70	90.84	99.6	99.6
8	baseball diamond	62.2	83.6	86.9	88.51	88.66	92.7	94.3
9	harbor	74.4	76.0	89.4	89.86	89.88	96.2	96.7
10	vehicle	65.1	46.1	84.0	90.63	90.82	97.0	97.7
11	crossroad	43.4	67.1	88.6	90.19	90.03	95.2	95.2
12	T junction	46.8	39.2	75.1	75.85	85.30	86.2	85.6
13	parking lot	45.8	37.5	63.3	69.44	75.36	75.8	76.0
	MAP	65.8	66.5	81.5	85.88	87.73	92.9	93.4

**Table 4:** Comparative experimental results of MFE on the NWPU VHR-10 Data set. (Rate: %).

Label	Name	Fast R-CNN	Mask R-CNN	RetinaNet	FCOS	CenterNet	SGFTHR	YOLOv7	MFE
1	airplane	78.01	80.83	78.10	77.84	75.94	78.12	99.5	99.5
2	ship	66.30	67.02	65.60	66.89	65.70	67.18	68.8	76.4
3	storage tank	84.88	84.22	84.85	85.03	83.12	86.49	67.7	77.9
4	tennis court	76.57	87.71	66.89	81.75	72.75	85.81	99.5	99.3
5	basketball court	43.38	45.73	44.85	40.19	43.86	46.15	99.6	99.3
6	ground track field	86.68	85.74	88.77	87.19	86.98	87.45	94.2	93.6
7	harbor	36.64	36.80	35.60	37.54	35.40	37.91	97.4	97.1
8	bridge	49.00	47.64	52.67	49.58	48.59	50.99	91.7	92.3
9	vehicle	79.73	76.91	68.89	87.35	72.89	93.33	98.2	98.8
10	baseball diamond	95.15	97.06	96.84	94.16	92.54	97.49	95.5	96.4
	MAP	69.64	70.97	68.31	70.75	67.78	73.10	91.2	93.1

**Table 5:** Comparative experimental results of DP-MLP on the TGRS-HRRSD-Dataset. (Rate: %).

Label	Name	Mask R-CNN	RS-YoloX	AFDet	Yolo-v4	Yolov5s	YOLOv7	DP-MLP
1	ship	89.6	90.1	92.0	90.18	89.3	95.0	95.7
2	bridge	87.6	90.0	89.1	90.47	60.2	94.3	95.2
3	ground track field	90.7	90.8	95.1	90.52	90.8	98.9	99.3
4	storage tank	89.9	90.3	96.0	90.39	97.0	96.1	97.5
5	basketball court	68.3	78.2	75.7	69.44	81.9	83.1	82.8
6	tennis court	90.3	90.4	93.9	90.34	96.0	97.0	96.8
7	airplane	90.8	90.9	98.4	90.70	98.9	99.6	99.7
8	baseball diamond	88.7	88.6	88.5	88.51	87.9	92.7	93.4
9	harbor	89.5	89.9	93.6	89.86	87.7	96.2	96.4
10	vehicle	90.1	90.8	96.2	90.63	98.0	97.0	97.8
11	crossroad	88.8	90.1	94.2	90.19	87.2	95.2	95.9
12	T junction	79.1	86.5	82.6	75.85	74.0	86.2	84.4
13	parking lot	66.5	75.0	65.4	69.44	58.1	75.8	75.8
	MAP	85.4	88.0	89.3	85.88	85.2	92.9	93.1

**Table 6:** Comparative experimental results of DP-MLP on the NWPU VHR-10 Data set. (Rate: %).

Label	Name	Mask R-CNN	RetinaNet	FCOS	CenterNet	SGFTHR	YOLOv7	DP-MLP
1	airplane	80.83	78.10	77.84	75.94	78.12	99.5	99.5
2	ship	67.02	65.60	66.89	65.70	67.18	68.8	73.3
3	storage tank	84.22	84.85	85.03	83.12	86.49	67.7	74.2
4	tennis court	87.71	66.89	81.75	72.75	85.81	99.5	99.4
5	basketball court	45.73	44.85	40.19	43.86	46.15	99.6	98.9
6	ground track field	85.74	88.77	87.19	86.98	87.45	94.2	93.1
7	harbor	36.80	35.60	37.54	35.40	37.91	97.4	97.9
8	bridge	47.64	52.67	49.58	48.59	50.99	91.7	90.0
9	vehicle	76.91	68.89	87.35	72.89	93.33	98.2	98.1
10	baseball diamond	97.06	96.84	94.16	92.54	97.49	95.5	96.9
	MAP	70.97	68.31	70.75	67.78	73.10	91.2	92.1

**Table 7:** Comparative experimental results on new datasets after generating images. Value unit: %.

Model	IOU = 0.50 (MAP)	IOU = 0.50:0.95 (MAP)
ARSL	80.4	48.4
YOLOv7	91.2	61.8
OURS	92.2	62.4

**Table 8:** Experimental results on the TGRS-HRRSD-Dataset. (Rate: %).

Label	Name	YOLOv7	MFE	DP-MLP
1	ship	95.0	95.8	95.7
2	bridge	94.3	95.2	95.2
3	ground track field	98.9	99.1	99.3
4	storage tank	96.1	97.2	97.5
5	basketball court	83.1	85.1	82.8
6	tennis court	97.0	97.3	96.8
7	airplane	99.6	99.6	99.7
8	baseball diamond	92.7	94.3	93.4
9	harbor	96.2	96.7	96.4
10	vehicle	97.0	97.7	97.8
11	crossroad	95.2	95.2	95.9
12	T junction	86.2	85.6	84.4
13	parking lot	75.8	76.0	75.8
	MAP	92.9	93.4	93.1

**Table 9:** Experimental results on the NWPU VHR-10 Dataset. (Rate: %).

Label	Name	YOLOv7	YOLOv8	MFE	DP-MLP	SSLM
1	airplane	99.5	86.4	99.5	99.5	99.9
2	ship	68.8	99.0	76.4	73.3	99.8
3	storage tank	67.7	86.5	77.9	74.2	69.7
4	tennis court	99.5	96.9	99.3	99.4	97.4
5	basketball court	99.6	85.2	99.3	98.9	99.0
6	ground track field	94.2	54.5	93.6	93.1	100
7	harbor	97.4	99.5	97.1	97.9	89.7
8	bridge	91.7	90.8	92.3	90.0	74.0
9	vehicle	98.2	67.9	98.8	98.1	92.9
10	baseball diamond	95.5	95.3	96.4	96.9	99.9
	MAP	91.2	86.4	93.1	92.1	92.2

**Table 10:** Comprehensive performance data sheet.

NO	Index	TGRS-HRRSD Dataset			NWPU VHR-10 Dataset				
		YOLOv7	MFE	DP-MLP	YOLOv7	YOLOv8	MFE	DP-MLP	SSLM
1	Layers	314	322	336	314	225	322	336	314
2	Parameters	73.6 MB	78.5 MB	119.3 MB	74.9 MB	43.67 MB	80.0 MB	77.0 MB	75.2 MB
3	FPS	149.3	127.3	125.0	149.3	75.8	112.4	87.7	138.2
4	FLOPs	104.7 G	108.4 G	109.7 G	104.7 G	89 G	107.1 G	115.6	108.9
5	mAP	92.9	93.4	93.1	91.2	86.4	93.1	92.1	92.2
6	P	91.1	91.8	92.4	90.4	90.5	94.1	96.0	92.1
7	R	88.7	90.1	89.9	89.0	80.7	87.6	86.8	89.7
8	F1	0.90	0.91	0.91	0.89	0.85	0.90	0.91	0.90

## Data Availability

Data are contained within the article.
